# Poor Effect of Empiric Antibiotic Treatment of Gram-Negative Bacteria on *Myroides* spp.: A Case Report and Literature Review

**DOI:** 10.1155/crdi/5745508

**Published:** 2025-10-03

**Authors:** Terhi Juusola, Marko Rahkonen, Elina Aho-Laukkanen, Vesa Mäki-Koivisto, Ilkka S. Junttila

**Affiliations:** ^1^Northern Finland Laboratory Centre (NordLab), Oulu 90220, Finland; ^2^Wellbeing Services County of Central Ostrobothnia, Kokkola 67101, Finland; ^3^Fimlab Laboratories, Tampere 33520, Finland; ^4^Faculty of Medicine and Health Technology, Tampere University, Tampere 33014, Finland; ^5^Research Unit of Biomedicine, University of Oulu, Oulu 90570, Finland

**Keywords:** bacteremia, case report, cat, chronic leg ulcers, empiric antibiotic treatment, *Myroides*, *Myroides odoratimimus*

## Abstract

**Background:**

*Myroides* spp. are a group of Gram-negative bacteria which may cause opportunistic infections in humans. They are widely resistant to clinically relevant empiric antibiotics. Importantly, the focus of infection, regional susceptibility data, and patient's medical history dictate the choice of an empiric antibiotic treatment. Unfortunately, the empiric treatment is not always optimal. Especially immunocompromised patients are at risk of having opportunistic infections, which are not necessarily common causes of typical infections.

**Case Presentation:**

We report here a case of a patient with several comorbidities, including chronic kidney failure, heart failure, and leg ulcers, who developed fever and infection of his chronic leg ulcers. Cefuroxime was initiated as an empiric treatment of suspected sepsis with unknown origin. Two days later, the initial blood culture sample indicated Gram-negative rods which VITEK MS mass spectrometry identified as *Myroides* spp. The patient's antibiotics were first changed to meropenem, and the next day, ciprofloxacin was added. After susceptibility testing, ciprofloxacin was changed to levofloxacin. Fortunately, the patient recovered with the treatment and was discharged from hospital.

**Conclusions:**

*Myroides* spp. are widely resistant opportunistic pathogens to acknowledge to be out of reach for first-line empiric antibiotic treatment. *Myroides* cases reported earlier have suggested soil exposure or pig bite as potential sources of infection. In the case described here, one possible source of *Myroides* might be cat scratches or cat licks.

## 1. Background

*Myroides* is a relatively poorly known genus of Gram-negative bacteria. PubMed search with words “*Myroides* infection” and filter “Humans” applied yields 45 publications (search day 2/9/2024). *Myroides* species (spp.) are Gram-negative, aerobic, nonmotile rods [1]. *Myroides* spp. are also catalase and oxidase positive [2, 3]. *Myroides* spp. were previously identified as *Flavobacterium odoratum*. On a nutrient agar, it appears yellow and produces a characteristic fruity odor [4]. *Myroides* spp. are common environmental bacteria in water and soil and only opportunistic pathogens in humans. They usually cause infections in immunocompromised patients, but there are also described cases in immunocompetent patients [1]. Initially it was considered that two *Myroides* species of the genus were clinically significant; these were *Myroides odoratus* and *Myroides odoratimimus* [5]. Recently, a third *Myroides* species, namely, *M. injenensis* has also been suggested to be a virulent one. Other *Myroides* species' clinical significance remains unclear [3].

Most commonly *Myroides* spp. cause cutaneous and soft-tissue infections, but also urinary tract infections, ventriculitis, endocarditis, and septicemia have been described [3, 6–10]. *Myroides* spp. are often resistant to multiple antibiotics [11]. Since its discovery as *Flavobacterium* in 1920, *Myroides* caused infections have been reported, with the number increasing in recent years [12]. This may in part be due to improved diagnostics methods, including matrix-assisted laser-desorption-ionization time of-flight (MALDI-TOF) mass spectrometry and nucleic acid–based methods (polymerase chain reaction [PCR] and sequencing).

*M. odoratus* and *M. odoratimimus* are reported to be resistant to a great number of antibiotics. In Gunzer et al.'s study [11], *Myroides* were tested widely resistant to penicillins, cephalosporins, and monobactams. Moxifloxacin was the most reliable agent of fluoroquinolones (54/59 susceptible), appearing more effective than ciprofloxacin (2/59) and levofloxacin (12/59), which was used in our case. Of carbapenems, the best option was meropenem (40/59 susceptible). The study used guidelines for PK/PD breakpoints according to the criteria published by the European Committee on Antimicrobial Susceptibility Testing (EUCAST). In O'Neal et al.'s [13] review, susceptibility results were rather similar, but only 26.2% of the reviewed cases were susceptible to meropenem. Interpretation was according to CLSI breakpoints for *Enterobacterales*. They considered minocycline (100.0%) and moxifloxacin (91.8%) as the most reliable agents against *Myroides* spp. Noteworthy was also that of 91 isolates tested, 0% was reported susceptible to colistin, which is used against multidrug-resistant Gram-negative rods. *M. odoratimimus* in our case showed a similar susceptibility pattern. In our case, cefuroxime was selected as an empiric antibiotic based on the National guidelines and recommendations. However, as described above, *Myroides* are widely resistant to cephalosporins. This highlights the importance of early identification of the causative pathogen to guide timely adjustment of antibiotic treatment in cases where initial empirical treatment is likely to be ineffective.

In most reported cases, the exact source of *Myroides* spp. has remained unclear, although they are common in soil and water [14]. For example, domestic dog scratches or pig bites have been suggested to be associated with *Myroides* infection [15, 16]. Also, exposure to soil, fresh water, or dog licks have been proposed to be a source of *Myroides* [12]. To our knowledge, no reports describing cats as a source for *Myroides* spp. infection in humans have been described. However, *Myroides* have been recognized as a part of subgingival microbial community in cats [17]. We describe here a case of *Myroides* spp. in a patient with several underlying medical conditions, leg ulcers, and domestic cats at home. Whether the infection originated from the cats remains unknown, but our case underlines the importance of recognizing *Myroides* spp. as a relatively antibiotic-resistant opportunistic pathogen.

## 2. Case Presentation

An 80-year-old male living in a house with multiple cats was referred to hospital emergency room (ER) by home care due to fever and acute deterioration of his general condition. He had chronic atrial fibrillation, diastolic heart failure, and chronic kidney failure (glomerular filtration rate, GFR 40 mL/min) which all together led to chronic swelling of both lower limbs and chronic leg ulcers. His weight was 120 kg (body mass index, BMI 39 kg/m^2^). He did not have a diagnosed immunosuppressive disease or medication, though his age and several comorbidities likely led to immunosenescence and impaired immune defense. His medication upon arrival to ER were beta blocker bisoprolol 5 mg × 1, Angiotensin-2 receptor blocker losartan 12.5 mg × 1, loop diuretic furosemide 40 mg 2 + 1, and anticoagulant apixaban 2.5 mg × 2.

In 2020, he had streptococcal bacteremia, spondylodiscitis, and epidural abscess treated with g-penicillin 5 MIU × 4 and clindamycin 600 mg × 3/day intravenously for four weeks following four weeks of clindamycin and levofloxacin 500 m mg × 1. From June 2023, his leg ulcers started to bother, and he had multiple antibiotic treatments including cefalexin and latest amoxicillin clavulanic acid 500/125 mg × 3/day ending a month before the leg ulcers became infected again.

The day before being admitted to ER in October 2023, the patient started to have a fever and a weakening general health. He had a home care visiting him a few times every week, and the next day they checked his C-reactive protein (CRP, 158 mg/L, reference value, rv, < 3) and referred him to ER. In the ER, the patient's health status was rather good, auscultation of the heart and the lungs were normal with irregular heart rate due to AF, the stomach was soft and painless, patient's hemodynamics were stable, blood pressure was 101/46 mmHg, heart rate 87/min, Glasgow Coma Scale 15, and body temperature normal. Both his legs had chronic swelling and increased redness. Thorax X-ray did not show signs of acute pneumonia. Influenza A/B, respiratory syncytial virus (RSV), and SARS-CoV-2 were tested negative by PCR. There were no bacteria in urine culture. CRP was 180 mg/L, leukocyte count 11.6 E9/L (rv 3.4–8.2 × 10^9^/L), hemoglobin 120 g/L (rv 134–167 g/L), creatinine 292 μmol/L (GFR 16 mL/min, rv 60–100, μmol/L, > 90 mL/min) suggesting dehydration due to infection, and blood sugar level 10.4 mmol/L (rv 4.2–6.0 mmol/L).

**Figure 1:**
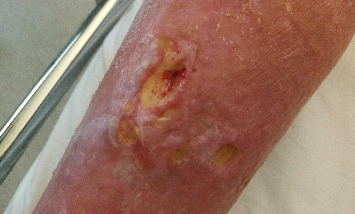
Swelling and secretion of the patients' chronic leg ulcers on 1.11.2023.

Cefuroxime 1.5 g × 2 was started intravenously as an antibiotic treatment with sepsis suspected as an acute diagnosis, following the national antibiotic guidelines. The patient was transferred to an internal medicine ward. The patient's condition stayed stable. He had mild fever (Table 1), leg ulcers were secreting serosa fluid (Figure 1), lower limbs were widely swollen below the knees, and his weight was increased from 119 kg to 126 kg. On his third day, at the ward, the blood culture was positive showing Gram-negative rods on microscopic examination. Later that day, *Myroides* sp. was first identified with VITEK MS mass spectrometry (MALDI-TOF, BioMérieux, France). Cefuroxime was discontinued as *Myroides* sp. is known to be widely resistant to cephalosporins, and intravenous meropenem 1 g twice a day was initiated. The 16S sequencing later confirmed the genus, and species was identified as *M. odoratimimus*.

The next day, the patient's fourth day in the ward, a specialist in infectious diseases examined the patient. The patient's legs were swollen and red referring to cellulitis (Figure 1). Some of the leg ulcers were moist and secreting. There were also scratch marks in thighs and upper parts of the shins, some of which might have been suitable for cat scratches. The chronic ulcers in the lower limbs were likely the source of the infection. Meropenem was continued, and intravenous ciprofloxacin 400 mg daily was added to the treatment.

On the patient's sixth day in the ward, he no longer had fever, and CRP and leukocytes had decreased (Table 1). A dermatologist also examined him and diagnosed stasis dermatitis from feet to knees and multiple ulcerations sizes of 1–3 cm (Figure 1). There was no sign of vasculitis or other rare cause of ulcers. The patient's toes were pale, and ATP and ADP were unpalpable but detected with Doppler. The dermatologist recommended topical treatment, Mepilex Ag, and corticosteroid cream daily. Bacterial culture of the ulcers was taken, resulting to *Staphylococcus aureus* and mixed aerobic bacteria; however, no *Myroides* was detected.

Further susceptibility testing of *Myroides* isolated from the blood cultures was performed. Ciprofloxacin MIC value was tested 1 mg/L and compared to breakpoints of other Gram-negative and catalase and oxidase positive group *Pseudomonas* spp. and PK-PD (nonspecies-related) 13.1 Breakpoint tables in EUCAST suggested it was resistant to ciprofloxacin. With the same criteria, levofloxacin (0.75 mg/L) was considered to be susceptible with a high dose (Table 2), and the patient's antibiotic was changed to intravenous levofloxacin 500 mg daily (reduced dose due to impaired kidney function), and meropenem was continued. Treatment was planned to continue for 14 days.

The general condition of the patient improved. The ulcers secreted less, and swelling reduced with topical treatment, bandages, and diuretics. On his ninth day in the ward, he had a fever of 37.7°Celsius again and was tested positive for SARS-CoV-2. He continued rehabilitation at a general medicine ward. He started gaining back his strengths and was discharged after a month from the beginning of this episode. Home care continued to treat his leg ulcers topically and with corticosteroid cream.

Investigations of a source of *Myroides* infection revealed that the patient had domestic cats with an access to outdoors. Due to cold weather conditions before hospitalization and the poor general condition of the patient, there was no direct contact of patient to outdoors as, for example, in the case described by Meyer et al. [12]. However, there was direct contact with the cats, and since they had direct access to the chronic ulcers of the patient, they could have been the possible origin of *Myroides* sp., either directly from the cats or transferred by the cats from the soil. *Myroides* sp. did not grow in the bacterial culture of the ulcers after the blood cultures were positive. Instead of *Myroides* sp., the bacterial culture contained *S. aureus* and mixed aerobic bacteria including *Serratia marcescens*.

## 3. Discussion and Conclusions

Empiric antibiotic treatment is chosen based on patient history, Gram stain results, and regional data of antibiotic resistance situation [18]. Currently, in Oulu University Hospital, cefuroxime is the first recommended antimicrobial treatment for home-acquired bacteremia against Gram-negative rods [19]. However, some of the Gram-negative rods are resistant to cefuroxime as our case report suggests. We previously reported a similar issue with *Francisella tularensis* [20]. It is important to take this into account when Gram stain shows Gram negative rod, or the patient's condition does not improve during antibiotic treatment. Especially, immunocompromised patients may have opportunistic bacteria causing sepsis [21].

Empiric antibiotic treatment also differs a lot depending on regional data of antibiotic resistance. Therefore, it is difficult to compare empiric treatments between different countries. Even within Nordic countries, where the resistance level is relatively similar and low compared to other European countries, empiric treatments vary. Finland was the only country recommending 2nd generation cephalosporin monotherapy to sepsis of unknown origin. Sweden, Norway, and Iceland recommended 3rd degree cephalosporins. In detail, Norway and Denmark recommend as an option a combination of benzylpenicillin or ampicillin and aminoglycoside and Sweden cefotaxime or piperacillin-tazobactam and aminoglycoside. All abovementioned countries had piperacillin-tazobactam as an option [22].

In the literature, *Myroides* spp. have been successfully treated with meropenem, piperacillin-tazobactam, ciprofloxacin, tigecycline, and levofloxacin depending on susceptibility data [6, 8, 9, 12, 23]. Our patient was treated with meropenem and levofloxacin after antimicrobial susceptibility testing (Table 1). The abovementioned empiric treatments of the Nordic countries (2nd or 3rd degree cephalosporins, or piperacillin-tazobactam) would seldomly cure *Myroides* infection, and therefore susceptibility testing is essential. Interpretation of susceptibility testing results is challenging for bacteria without specific breakpoints. In Table 2, we used EUCAST PK/PD breakpoints and breakpoints for another oxidase and catalase positive rod, *Pseudomonas aeruginosa.* Both ways have their limitations to directly utilize the interpretations to practice.

*Myroides* spp. are commonly found in the environment, but their role in human microbiota is uncertain. They are inherently resistant to many antibiotics. Recent study on *Myroides* antibiotic resistance indicated that 51 *M. odoratimimus* that were multidrug resistant, possessed capacity of biofilm formation and contained several genomic virulence factors [24]. The combination of these factors leads to a considerable challenge for the effective antibiotic treatment of *Myroides* infections. Our case indicates the importance of acknowledging *Myroides* as a cause of relatively antibiotic-resistant infection in a geographical area of relatively low general antibiotic resistance.

Few cases of *Myroides*-caused urinary tract infection outbreaks have been reported in hospitals. Usually, the patients of these outbreaks had underlying conditions and other risk factors, such as intensive care, a long hospitalization, catheters, use of broad-spectrum antibiotics, or diabetes [9, 25]. In Ktari et al.'s study [26], seven patients had *M. odoratimimus* urinary tract infections. Six of them had urinary calculi and endourological surgery, and all the patients had a prolonged hospital stay as a risk factor. Also, Khan et al. [27] reported five catheter-related *Myroides*-caused urinary tract infections, sensitive only to minocycline. Considering these outbreaks and the biological features of *Myroides,* it may be important bacteria to acknowledge in future hospital hygiene protocols, and whether *Myroides* should be actively screened in these instances will be worth further studies.

The risk factors of our patient for developing an opportunistic infection were old age, several illnesses, and chronic leg ulcers. Fortunately, he recovered from the infection with the correct antibiotic treatment, which was escalated from an ineffective empiric antibiotic after pathogen naming and again after susceptibility testing. The patient had domestic cats with access to outdoors and possible cat scratches near his leg ulcers. The cats might have transferred *Myroides* from soil to the patient by scratching, or they might have infected the ulcers directly from their oral microbiota [17] by licking the ulcers. Recently, Rodrigues et al. [17] found *Myroides* sp. from the oral microbiota of healthy cats by next-generation sequencing. The role of the cats as the source of *Myroides* can neither be confirmed nor can it be ruled out. Further research on this topic is needed.

Our case underlines the importance of continuous evaluation of efficiency of an empiric antibiotic treatment. In this context, the role of clinical laboratory is to support clinician in this work by efficiently providing rapid identification of causative microbe and providing some tools as regard to antimicrobial sensitivity of the causative microbe.

## Figures and Tables

**Table 1:** Fever, leukocytes, and CRP.

Date and antibiotic treatment	CRP (rv < 3 mg/L)	Fever (rv∼37°C)	Leukocyte count (rv 3.4–8.2 × 10^9^/L)
26.10.			
27.10. Cefuroxime	180	38.0	11.6
28.10. Cefuroxime	208	37.6	
29.10. Meropenem	176	37.6	10.0
30.10. Meropenem and ciprofloxacin	106	37.0	7.1
31.10. Meropenem and ciprofloxacin			
1.11. Meropenem and levofloxacin	75		6.9
2.11. Meropenem and levofloxacin			
3.11. Meropenem and levofloxacin			
4.11. Meropenem and levofloxacin			
5.11. Meropenem and levofloxacin			
6.11. Meropenem and levofloxacin		37.7	
7.11. Meropenem and levofloxacin			
8.11. Meropenem and levofloxacin			
9.11. Meropenem and levofloxacin			
10.11. Meropenem and levofloxacin			
11.11. Meropenem and levofloxacin			
12.11. Meropenem and levofloxacin			
13.11. Meropenem and levofloxacin			
14.11. Meropenem and levofloxacin			
15.11. End of treatment	14		7.3

*Note:* The patients' fever (Celsius), leucocytes (E9/L), and CRP (mg/L) during antibiotic treatment. The empty cells indicate that data were not available for those days.

**Table 2:** Susceptibility testing.

Antibiotics	Our disc	Our MIC	Interpretation with *Pseudomonas* spp. breakpoints (MIC and disc)	Interpretation with EUCAST 13.1 PK-PD breakpoints (only MICs)
Amikacin		> 32 = R	R	R
Amoxicillin-clavulanic acid	13 (NR)	16 = R	NA	R
Aztreonam	10 (NR)	32 = R	R	R
Ertapenem	23 (NR)	3 = ?	NA	R
Imipenem		32 = R	R	R
Ceftolozane-tazobactam		32 = R	R	R
Cefotaxime	(NR)	> 8 = R	NA	R
Ceftazidime-avibactam		> 16 = R	R	R
Ceftazidime	6 = R	> 16 = R	R	R
Ceftriaxone	6 = R (NR)		NA	
Cefuroxime	6 = R		NA	
Colistin		> 8 = R	R	IE
Levofloxacin		0.75 = S^∗^	I	I
Meropenem	16 = ?	6 = R	I	I
Piperacillin-tazobactam	10 = R		R	
Ciprofloxacin	23 = ?	1 = R	R	R
Trimethoprim-sulfamethoxazole	10 = R (NR)	0.75 = S	NA	IE
Tigecycline		1 = ?	NA	R
Tobramycin	6 = R	> 8 = R	R	R

*Note:* NR = not reported to clinic, I = intermediate, S = susceptible, and R = resistant.

Abbreviations: IE = insufficient evidence and NA = not available.

^∗^Levofloxacin was reported as S, after advising the clinician of the requirement of higher levofloxacin dosage, and the patient was treated with intravenous levofloxacin 500 mg daily (reduced for kidney function).

## Data Availability

Data sharing is not applicable to this article as no new data were created or analyzed in this study.

## Nomenclature

ER: Emergency room
EUCAST: The European Committee on Antimicrobial Susceptibility Testing
